# Complete Genome Sequence of the Exopolysaccharide-Producing *Burkholderia caribensis* Type Strain MWAP64

**DOI:** 10.1128/genomeA.01636-15

**Published:** 2016-01-28

**Authors:** Yanling Pan, Ka Fai Kong, Jimmy S. H. Tsang

**Affiliations:** Molecular Microbiology Laboratory, School of Biological Sciences, University of Hong Kong, Hong Kong Special Administrative Region, China

## Abstract

We report the complete genome sequence of *Burkholderia caribensis* MWAP64 (LMG 18531), which was isolated from soil for its proficiency in producing large amounts of exopolysaccharide that help form microaggregates in a vertisol. There are four replicons with a total size of 9,032,119 bp.

## GENOME ANNOUNCEMENT

*Burkholderia caribensis* MWAP64 (LMG 18531) was isolated from a fraction of a vertisol in the southeast of the island of Martinique in the French West Indies (1). It is a motile, Gram-negative, rod-shaped bacterium that is pleomorphic in actively growing cultures. It produces large amounts of exopolysaccharide in sugar-enriched agar media and forms mucoid colonies. It is the type strain of this species. Here, we describe the complete genome sequence of this bacterium.

Previous studies on a haloacid-degrading bacterium strain MBA4 using DNA-DNA hybridization showed that it has a DNA homology value of 74% with that of *Burkholderia caribensis* MWAP64 (2). The complete genome of MBA4 was recently determined and deposited in GenBank as CP012746, CP012747, and CP012748 (3). For strain MWAP64, a whole-genome shotgun approach was used with the Roche 454 GS FLX Titanium and PacBio RS II systems. The raw sequencing reads of MWAP64 obtained from the 454 sequencing platform were filtered and analyzed with CLC Genomic Workbench version 6.5 (CLC bio, Aarhus, Denmark). There were 138,033 clean reads with an average length of 355 bp. For the PacBio long-read sequencing system, SMRT Analysis version 2.3.0 and HGAP version 2 were used for filtering, trimming, and assembly. There were 73,629 clean reads with an average length of 11,521 bp. The depth of coverage is around 100-fold. The genome of *B. caribensis* MBA4 was used as a reference sequence. More than 99.94% of the 48,990,523 bp from the 454 clean reads were mapped to the assembled contigs. Four circular replicons, two chromosomes, and two plasmids were assembled. The sizes of these four replicons are, respectively, 3,688,127; 2,890,272; 2,011,268; and 442,452 bp. The total size of the genome is 9,032,119 bp, with a tabulated GC content of 62.58%.

The completed genome was annotated automatically with the Prokaryotic Genomes Automatic Annotation Pipeline (PGAAP) from NCBI (4) and the Rapid Annotations using Subsystems Technology (RAST) server (5). The complete genome contains 7,911 predicted genes, including 18 rRNAs, 62 tRNAs, and 1 ncRNA. Apart from 157 pseudogenes, the genome contains 7,673 protein-coding genes. Among these protein-coding genes, 5,768 were assigned with functions, while 1,905 were annotated as hypothetical. Furthermore, 634 tandem repeats were identified by Tandem Repeats Finder (6). The online CRISPR finder (7) predicted the presence of 7 questionable CRISPR regions in the genome. The online prophage search tool PHAST (8) identified one intact, one incomplete, and one questionable prophage region in the four replicons. The metagenomic analysis server WebMGA (9) assigned 5,946 genes into 23 clusters of orthologous groups (COG) categories. Pathway Tools version 19.5 predicted 283 pathways, including 1,647 enzymatic reactions and 17 transport reactions (10).

### Nucleotide sequence accession numbers.

The genome sequence of *Burkholderia caribensis* MWAP64 has been deposited in GenBank under the accession numbers CP013102, CP013103, CP013104, and CP013105.
